# Bibliometric and visualized analysis of nonpharmaceutical TCM therapies for rheumatoid arthritis over the last 20 years using VOSviewer and CiteSpace software

**DOI:** 10.1097/MD.0000000000035305

**Published:** 2023-09-29

**Authors:** Xiaojun Sun, Hongqin Yin, Yanhui Zhu, Ling Li, Jun Shen, Kongfa Hu

**Affiliations:** a Traditional Chinese Medicine Inheritance and Innovation Center, Taizhou Hospital of TCM Affiliated to Nanjing University of Chinese Medicine, Taizhou, China; b Department of Acupuncture–Moxibustion and Tuina, Taizhou Hospital of TCM Affiliated to Nanjing University of Chinese Medicine, Taizhou, China; c Department of Orthopedics, Taizhou Hospital of TCM Affiliated to Nanjing University of Chinese Medicine, Taizhou, China; d Department of Rheumatology, Taizhou Hospital of TCM Affiliated to Nanjing University of Chinese Medicine, Taizhou, China; e Ward of Traditional Chinese Medicine Classics, Taizhou Hospital of TCM Affiliated to Nanjing University of Chinese Medicine, Taizhou, China; f School of Artificial Intelligence and Information Technology, Nanjing University of Chinese Medicine, Nanjing, China; g Jiangsu Collaborative Innovation Center of Traditional Chinese Medicine in Prevention and Treatment of Tumor, Nanjing, China.

**Keywords:** bibliometric analysis, nonpharmaceutical therapy, rheumatoid arthritis, traditional Chinese medicine, visualization

## Abstract

**Background:**

Rheumatoid arthritis (RA) is a chronic autoimmune inflammatory disease that poses a significant threat to a patient’s quality of life. Commonly used drugs include glucocorticoids, nonsteroidal anti-inflammatory drugs, disease-modifying antirheumatic drugs, and biological agents; however, there are associated side effects. Complementary and alternative medicines can play positive roles. Bibliometric analysis of herbal medicines for RA has been conducted, but current research trends in nonpharmaceutical traditional Chinese medicine (TCM) therapies for the treatment of RA have not been studied. Here, we conducted a bibliometric analysis of the application of nonpharmaceutical TCM therapies for RA over the last 20 years.

**Methods:**

We retrieved relevant literature from the Web of Science Core Collection database and used VOSviewer and CiteSpace software for analysis. Visualized maps were then generated to display the relationships between the author, country, institution, and keywords.

**Results:**

A total of 567 articles were included in the final analysis. The number of annual publications on nonpharmaceutical TCM interventions for RA increased over the study period. The journal with the highest number of publications on this topic was *Evidence-based Complementary and Alternative Medicine*; however, *Cochrane Database of Systematic Reviews* had the most citations. Collaborations were observed among worldwide institutions, with the People’s Republic of China playing a dominant role in the research on treatment of RA using nonpharmaceutical TCM therapies. Ernst E was the most productive author, with 11 articles, whereas Green S had the highest number of citations (287) at the time of retrieval. Specific improvements in the efficacy and selection of nonpharmaceutical therapies were the main research hotspots based on citation burst analysis.

**Conclusion:**

This study characterizes the trends in the literature for nonpharmaceutical TCM therapy for RA over the past 20 years; showcasing the current research status for relevant researchers and their teams and providing a reference for future research directions.

## 1. Introduction

Rheumatoid arthritis (RA) is a chronic autoimmune joint disease that affects approximately 1% of the global population, and is characterized by cartilage and bone tissue damage, synovial hyperplasia, chronic inflammation, and pannus formation.^[1,2]^ Patients often experience joint pain, deformity, and even disability due to loss of function.^[2]^ The incidence of the disease is significantly higher in women than in men,^[3]^ with the risk of lifetime illness in adult women being 3.6%.^[4]^ Patients with RA experience permanent damage and destruction of the bone and joint structures due to inflammation-induced cartilage and bone erosion caused. Therefore, without effective treatment, RA may lead to disability and loss of self-care and work abilities.^[5]^

As a chronic autoimmune disease, the pathogenesis of RA, which may be related to immune responses, inflammatory factors, matrix metalloproteinases, abnormal oxidative stress, genetics, and the living environment, has not been fully elucidated.^[6]^

Drugs commonly used to treat RA include glucocorticoids, nonsteroidal anti-inflammatory drugs, disease-modifying antirheumatic drugs (DMARDs), biological agents, and Chinese herbal medicines.^[7,8]^

Although medication may delay the progression and control most symptoms, RA cannot be cured. Long-term treatment is required, which cannot be performed in clinics and may have certain toxicity or side effects. Nonpharmaceutical therapies, such as acupuncture, electroacupuncture (EA), moxibustion, and Tai Chi are widely used in traditional Chinese medicine (TCM).^[9,10]^ In the treatment of RA, nonpharmaceutical TCM interventions have shown cost-efficient, safe, and long-lasting efficacy that has been well accepted.

The term “bibliometrics” refers to the quantitative evaluation of literature and is widely used to evaluate research trends and hotspots across many fields.^[11]^ The bibliometrics software VOSviewer is frequently used for coword and cocitation analyses in addition to bibliographic coupling. It can clearly display the results and help understand the research hotspots, knowledge mapping, collaborative relationships, and future directions of a certain field and provides a strong basis for developing clinical guidelines.^[12]^ Currently, this method is extensively used in various fields.^[13,14]^

In recent years, multiple studies have explored nonpharmaceutical TCM therapies for RA^[15,16]^; however, no systematic analysis has yet been conducted. Therefore, in this study, we used bibliometric software to systematically analyze and visualize studies on this topic published over the past 20 years (2002 to 2022). In addition, this study aimed to summarize the achievements in this field and reveal research directions and hotspots that can provide a reference for future studies.

## 2. Materials and methods

### 2.1. Searching strategy

Related publications were collected from the Science Citation Index Expanded (SCI-E) through the Web of Science Core Collection database (WOS; https://www.webofknowledge.com; accessed April 20th, 2023. The retrieval strategy was “(TS= [‘moxibustion’ OR ‘acupuncture’ OR ‘electroacupuncture’ OR ‘auricular points’ OR ‘needle’ OR ‘needling’ OR ‘embedding’ OR ‘acupoint’ OR ‘cupping’ OR ‘guasha’ OR ‘massage’ OR ‘Qigong’ OR ‘Tuina’ OR ‘Tai Chi’ OR ‘Wuqinxi’ OR ‘Baduanjin’ OR ‘Yijinjing’]) AND TS= (‘Rheumatoid arthritis’ OR ‘RA’)” (Table S1, Supplemental Digital Content, http://links.lww.com/MD/K63). The retrieval time span was from 2002 to 2022, and the literature types were set as “article” and “review.”

### 2.2. Data analysis

The included literature was exported in “Plain Text File,” and the titles, keywords, institutions, journals, countries or regions, authors, and references of each literature were retrieved. The VOSviewer software (version 1.6.17; Centre for Science and Technology Studies, Leiden University, Leiden, Netherlands), CiteSpace 5.7. R3 software (http://cluster.cis.drexel.edu/~cchen/citespace/download/), and Microsoft Excel 2020 were used for data analysis and visualization of nonpharmaceutical TCM therapies for RA. In this study, we did not collect individual patient data and hence ethical approval was not required.

Jointly developed by van Eck and Waltman of Leiden University in the Netherlands, VOSviewer is a bibliometric analysis tool used to draw knowledge maps. It can perform coword, cocitation, and bibliographic coupling analyses and provide a visualization of the research results. This method offers unique advantages for clustering and graphics display.^[17,18]^ CiteSpace is commonly used to detect and visualize current scientific knowledge, trends in literature, and identify future research directions.^[19]^ Microsoft Excel 2020 is used to construct relevant tables and display the yearly trends in publications and citations.

## 3. Results

**Figure 1.**
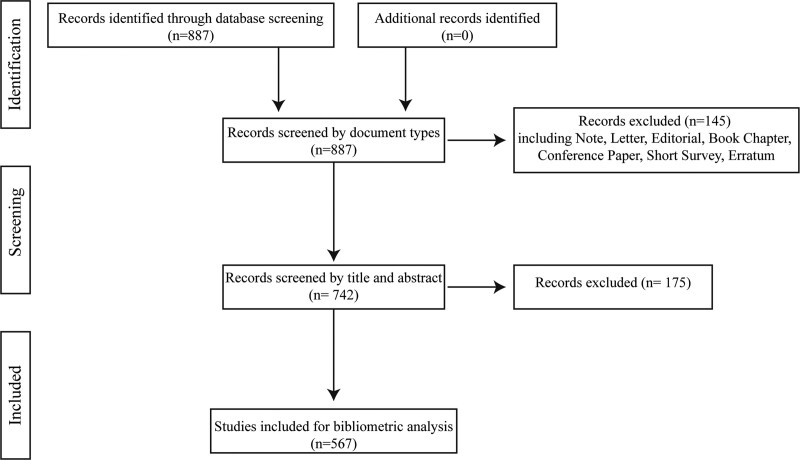
Flow diagram of the screening process.

In total, 887 relevant studies were retrieved. After excluding conference papers, editorials, letters, books, and duplicates, 567 articles were included in this study (Fig. 1).

### 3.1. Trends of publications and citations

Typically, the developmental status and future research trends in a field are reflected by changes in the number of annual publications and citations. As of December 2022, 567 articles on nonpharmaceutical TCM therapies for RA were included in the core collection of the WOS database (Fig. 1).

**Figure 2.**
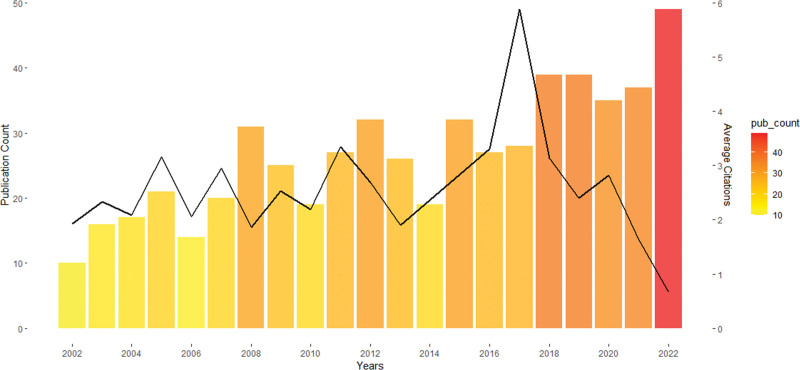
The annual publication count and annual average citations from 2002 to 2022.

From 2010 to 2020, the number of published articles on RA showed an overall increasing trend (Fig. 2). The average annual citation frequency reached its highest level (5.88) in 2017. Although the number of publications in 2022 was the highest (n = 49) of the past 20 years, the average annual citation frequency was only 0.67 (Fig. 2).

### 3.2. Characteristics of countries or regions and institutions

From 2002 to 2022, researchers from 51 countries or regions conducted studies on nonpharmaceutical TCM therapies for RA. The People’s Republic of China published the highest number of articles (147; 25.93%), followed by the USA (124; 21.87%) and South Korea (53; 9.35%). In terms of total citations, the USA (4519), People’s Republic of China (1710), and South Korea (1490) were ranked in the top 3; however, Australia (61.73), the United Kingdom (43.39), and the USA (37.97) had higher average citations (Table 1). These findings suggested that the People’s Republic of China, South Korea, and the USA have significant influence in this field.

**Table 1:** Publications and citations in top 10 countries or regions.

Rank	Country	Publication	% of 567	Country	Total citation	Average citation
1	People’s Republic of China	147	25.93	USA	4519	37.97
2	USA	124	21.87	People’s Republic of China	1710	11.1
3	South Korea	53	9.35	South Korea	1490	31.04
4	United Kingdom	37	6.53	United Kingdom	1432	43.39
5	Germany	29	5.11	Australia	679	61.73
6	Australia	24	4.23	Germany	497	19.88
7	Canada	22	3.88	Netherlands	492	35.14
8	Netherlands	13	2.29	Canada	375	23.44
9	Egypt	12	2.12	India	198	24.75
10	Brazil	9	1.59	Switzerland	184	36.8

A total of 826 institutions conducted RA-related studies using nonpharmaceutical TCM therapies. Among these, Kyung Hee University (45 articles), Chengdu University of Traditional Chinese Medicine (20 articles), and The University of Maryland (20 articles) were the 3 most influential institutions in this area. In addition, the People’s Republic of China accounted for 7 of the top ten institutions in terms of publication count, indicating its leading role in this research area (Table 2). Detailed citation analysis of countries and institutions can be found in Figure S1, Supplemental Digital Content, http://links.lww.com/MD/K64.

**Table 2:** Distribution of top 10 institutions in terms of publication count.

Rank	Institution	Record (n)	Average citation	H-index^[20]^	Original country
1	Kyung Hee Univ	45	35.96	17	South Korea
2	Chengdu Univ Tradit Chinese Med	20	4.38	7	People’s Republic of China
3	Univ Maryland	20	42.35	12	USA
4	Shanghai Univ Tradit Chinese Med	19	13.73	7	People’s Republic of China
5	Sun Yat Sen Univ	19	18	8	People’s Republic of China
6	Nanjing Univ Chinese Med	17	10.22	5	People’s Republic of China
7	China Med Univ	16	16	5	People’s Republic of China
8	Zhejiang Chinese Med Univ	16	12.2	6	People’s Republic of China
9	Tianjin Univ Tradit Chinese Med	15	12	5	People’s Republic of China
10	Tufts Univ	15	23.38	8	USA

The H-index measures the number of papers to which a researcher is a coauthor and the number of times each paper has been cited.

### 3.3. Academic collaborations

**Figure 3.**
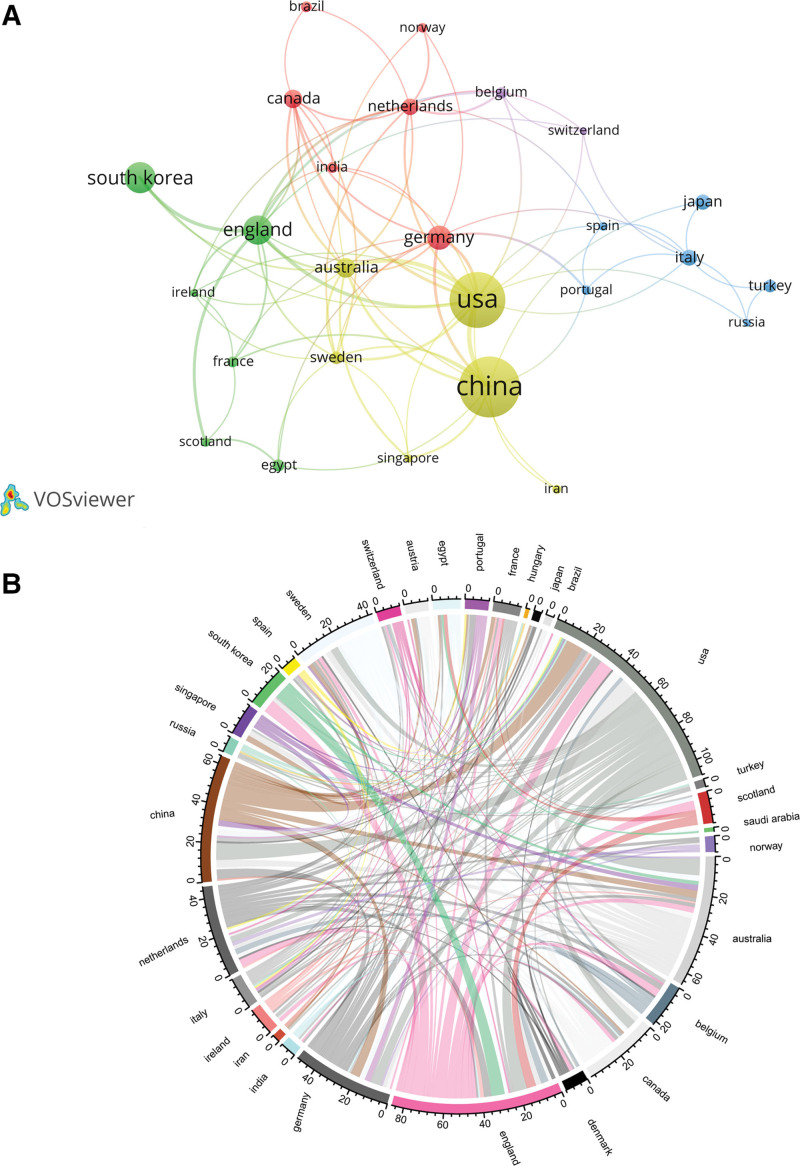
Map of countries or regions with cooccurrence networks. (A) Academic collaborations between countries or regions with more than 5 publications. The node size indicates the number of articles published by countries or regions; thickness of lines represents collaboration strength; different colors represent different collaboration groups. (B) The top 25 countries or regions with academic collaboration. Line thickness between countries or regions reflects the frequency of interaction.

**Figure 4.**
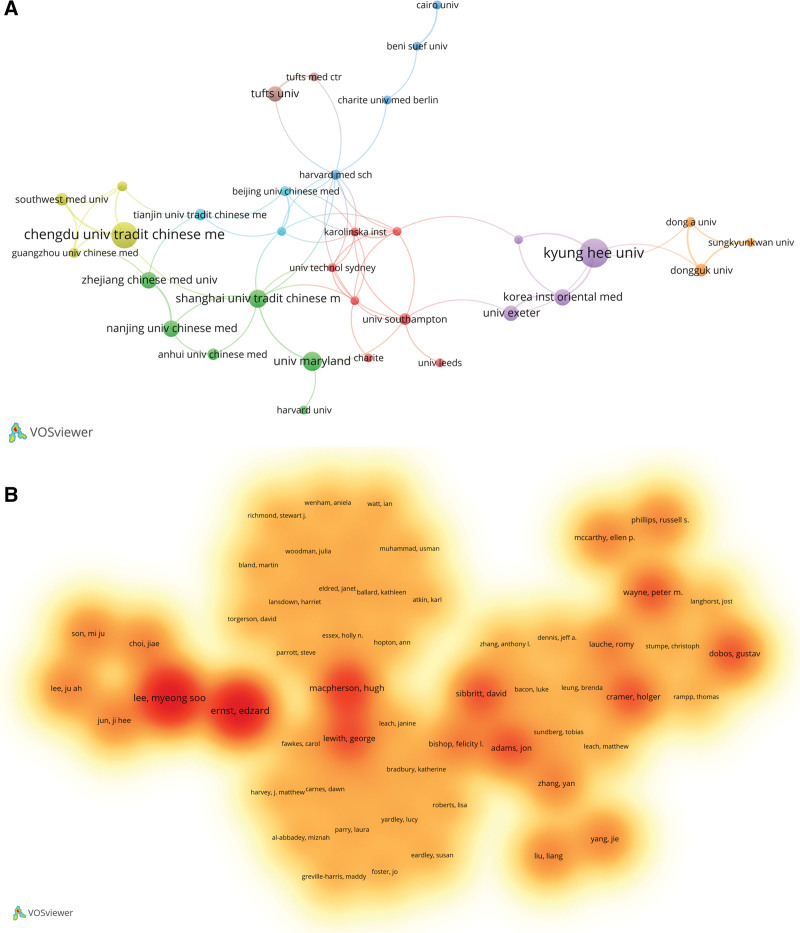
Mapping of collaborative institutions and authors. (A) Collaboration between different institutions. Circles or nodes in different colors represent different collaborative groups. (B) Collaboration with different authors.

Academic exchanges and collaborations among various countries, regions, institutions, and authors are conducive to the deep exploration of specific research areas. This concept was also applicable to our study, and multilevel collaborations among different institutions were observed (Figs. 3 and 4).

As shown in Figure 3A, 26 countries or regions published at least 5 research papers each. Figure 3B shows that the USA, People’s Republic of China, United Kingdom, and Germany had the highest number of collaborations with other countries or regions.

Collaboration between institutions reflects closeness. Interestingly, 40 institutions published at least 5 articles each. Different-colored circles or nodes represent the collaborative groups, as shown in Figure 4A. Among them, Kyung Hee University, Chengdu University of Traditional Chinese Medicine, and University of Maryland had the highest number of collaborations with other institutions. Further analysis of 2828 coauthors revealed that Ernst E (11 articles) and Lee MS (11 articles) were the most productive researchers. The academic influence of authors was evaluated using the H-index, revealing Ernst E (H-index = 11), Lee MS (H-index = 10), and Kim KS (H-index = 8) as the top 3 most influencing authors (Table 3). Clustering showed that a total of 57 authors collaborated closely (Fig. 4B) with Ernst E and Lee MS showing the greatest significance in this collaborative network and indicating the extensive collaboration of these authors with others.

**Table 3:** Distribution of top ten authors by publication count, citations, and H-index.

Rank	Author	Publication count	Cited author	Total citations	Highly H-index author	H-index
1	Ernst E	11	Green S	287	Ernst E	11
2	Lee MS	11	Buchbinder R	287	Lee MS	10
3	Adly AS	8	Hetrick S	287	Kim KS	8
4	Kim KS	8	Astin JA	275	Kim CH	7
5	Liu L	8	Eisenberg DM	275	Lee YC	7
6	Wang CC	8	Forys KL	275	Chen Y	6
7	XU X	8	Shapiro SL	275	Lao LX	6
8	Kim CH	7	Lee JD	257	Li J	6
9	Lao LX	7	Lee MS	253	Wang CC	6
10	Lee YC	7	Wang CC	246	Chang YC	5

Further analysis of the country and academic institutions with the highest number of collaborations demonstrated that the People’s Republic of China and its domestic academic institutions played a dominant role in the research on nonpharmaceutical TCM therapies for RA.

### 3.4. Distribution of cocitation authors

**Figure 5.**
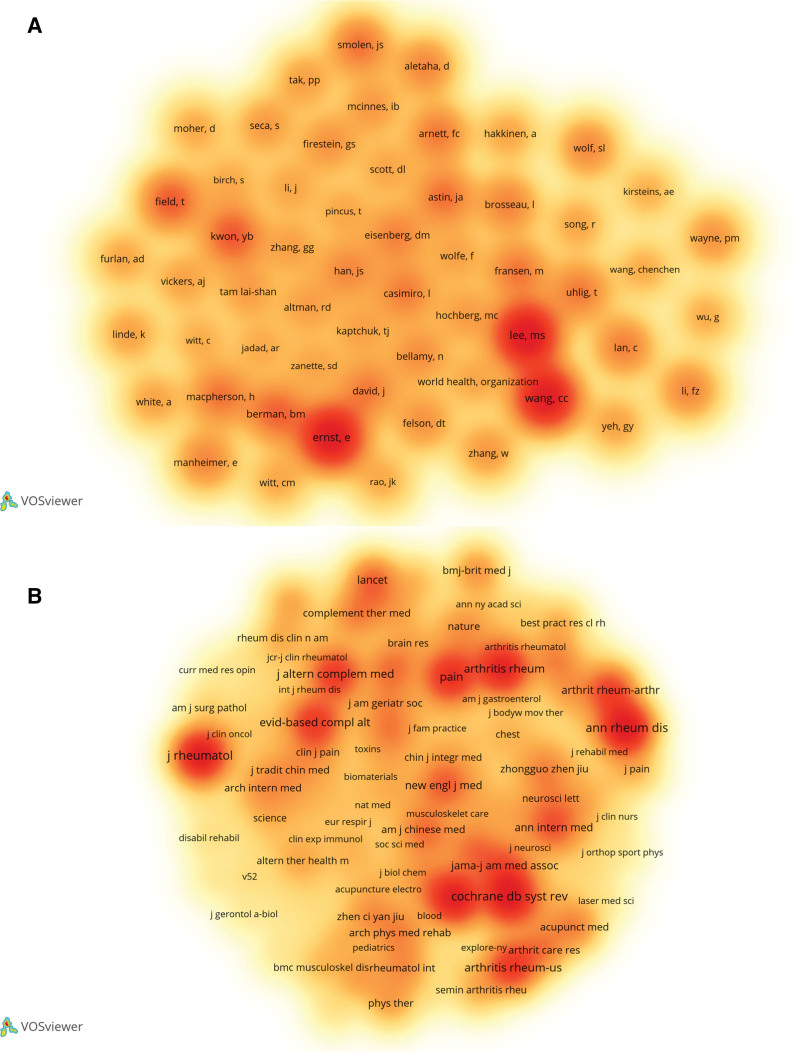
Visualization of cocitation authors and journals using VOSviewer. (A) Cocitation authors. (B) Cocitation journals.

Cocitation refers to 2 published articles citing earlier publications. Figure 5A shows that author Lee MS had the highest number of cocitations (120), followed by Ernst E (116) and Wang CC (114), indicating that their contributions to this field have attracted widespread attention.

### 3.5. Journal distribution

Further analysis showed that relevant articles were published in 292 journals, with 29 of these journals publishing more than 5 articles related to nonpharmaceutical TCM therapies for RA. Journal quartiles were based on journal citation reports from 2021. The top 4 high-yield journals were *Evidence-based Complementary and Alternative Medicine* (Q3), *Journal of Alternative and Complementary Medicine* (Q3), *Medicine* (Q3), and *Rheumatology* (Q1). The top 4 cited journals were *Cochrane Database of System Reviews* (Q1), *Evidence-based Complementary and Alternative Medicine* (Q3), *Rheumatology* (Q1), and *Journal of Alternative and Complementary Medicine* (Q3). The top 3 journals in terms of the H-index were *Evidence-based Complementary and Alternative Medicine* (Q3), *Rheumatology* (Q1), and *Journal of Alternative and Complementary Medicine* (Q3). Notably, published articles and the top ten cited journals were mostly distributed in the Q1 or Q3 regions (Table 4), indicating the importance of these journals for research on nonpharmaceutical TCM therapies for RA.

**Table 4:** Distribution of top ten journals by publication count, citations, and H-index.

Rank	Journal	Publication count	% of 567	JCR quartile	Journal	Total Citations	JCR quartile	Journal	H-index	JCR quartile
1	*Evidence-based Complementary and Alternative Medicine*	30	5.29	Q3	*Cochrane Database of Systematic Reviews*	1140	Q1	*Evidence-based Complementary and Alternative Medicine*	13	Q3
2	*Journal of Alternative and Complementary Medicine*	12	2.12	Q3	*Evidence-based Complementary and Alternative Medicine*	808	Q3	*Rheumatology*	11	Q1
3	*Medicine*	11	1.94	Q3	*Rheumatology*	349	Q1	*Journal of Alternative and Complementary Medicine*	10	Q3
4	*Rheumatology*	11	1.94	Q1	*Journal of Alternative and Complementary Medicine*	292	Q3	*American Journal of Chinese Medicine*	8	Q1
5	*American Journal of Chinese Medicine*	10	1.76	Q1	*American Journal of Chinese Medicine*	268	Q1	*Journal of Rheumatology*	8	Q2
6	*Rheumatology International*	9	1.59	Q3	*Rheumatology International*	230	Q3	*Rheumatology International*	8	Q3
7	*Acupuncture in Medicine*	8	1.41	Q3	*Journal of Rheumatology*	207	Q2	*Acupuncture in Medicine*	7	Q3
8	*Journal of Acupuncture and Tuina Science*	8	1.41	Q4	*Annals of The Rheumatic Diseases*	202	Q1	*Annals of The Rheumatic Diseases*	6	Q1
9	*Journal of Rheumatology*	8	1.41	Q2	*Complementary Therapies in Clinical Practice*	191	Q2	*Clinical Rheumatology*	6	Q3
10	*Clinical Rheumatology*	7	1.23	Q3	*Chinese Journal of Integrative Medicine*	182	Q3	*Cochrane Database of Systematic Reviews*	6	Q1

*Notes*: Journal quartile and H-index refer to journal citation reports from 2021.

JCR = journal citation report, Q = quartile.

According to the analysis of cocitation journals, 177 of 5602 frequently cited journals published at least 20 studies. The top 5 journals were *Annals of the Rheumatic Diseases* (561 citations), *Journal of Rheumatology* (536 citations), *Cochrane Database of System Reviews* (520 citations), *Rheumatology* (478 citations), and *Arthritis Research & Therapy* (448 citations) (Fig. 5B).

### 3.6. Analysis of highly cited articles

As indicated in Table 5, one of the top 2 cited articles was “Acupuncture for shoulder pain” by Green S in 2005 (287 citations). This systematic review aimed to determine the efficacy and safety of acupuncture for the treatment of shoulder pain. It argued that owing to the limited number of clinical and methodological trials, there is little evidence to support or refute the use of acupuncture for the treatment of shoulder pain despite the short-term relief of pain and gain of function; therefore, it suggested that high-quality clinical trials are warranted.^[21]^ The other article was “Physical activity and exercise for chronic pain in adults: an overview of Cochrane Reviews” by Geneen LJ in 2017 (281 citations). This study aimed to evaluate the improvement in chronic pain following the performance of exercises such as Tai Chi. Studies have shown that physical activity and exercise can alleviate pain and improve body function with almost no side effects, thereby improving the quality of life.^[22]^ Both papers were published in the *Cochrane Database of System Reviews*, indicating the great importance of this journal in reporting nonpharmaceutical TCM therapies for RA.

**Table 5:** Top 10 cited articles.

Rank	Author	Title	Source	Citations
1	Green S, 2005	Acupuncture for shoulder pain	*Cochrane Database of Systematic Reviews*	287
2	Geneen LJ, 2017	Physical activity and exercise for chronic pain in adults: an overview of Cochrane Reviews	*Cochrane Database of Systematic Reviews*	281
3	Astin JA, 2003	Mind-body medicine: state of the science, implications for practice	*Journal of The American Board of Family Practice*	275
4	Sinusas K, 2012	Osteoarthritis: diagnosis and treatment	*American Family Physician*	224
5	Zijlstra FJ, 2003	Anti-inflammatory actions of acupuncture	*Mediators of Inflammation*	199
6	Fransen M, 2008	Exercise for osteoarthritis of the knee	*Cochrane Database of Systematic Reviews*	191
7	Wasserman AM, 2011	Diagnosis and management of rheumatoid arthritis	*American Family Physician*	159
8	Kavoussi B, 2007	The neuroimmune basis of anti-inflammatory acupuncture	*Integrative Cancer Therapies*	157
9	Yeh GY, 2011	Tai Chi exercise in patients with chronic heart failure a randomized clinical trial	*Archives of Internal Medicine*	154
10	Morone NE, 2007	Mind–body interventions for chronic pain in older adults: a structured review	*Pain Medicine*	150

### 3.7. Analysis of keywords

**Figure 6.**
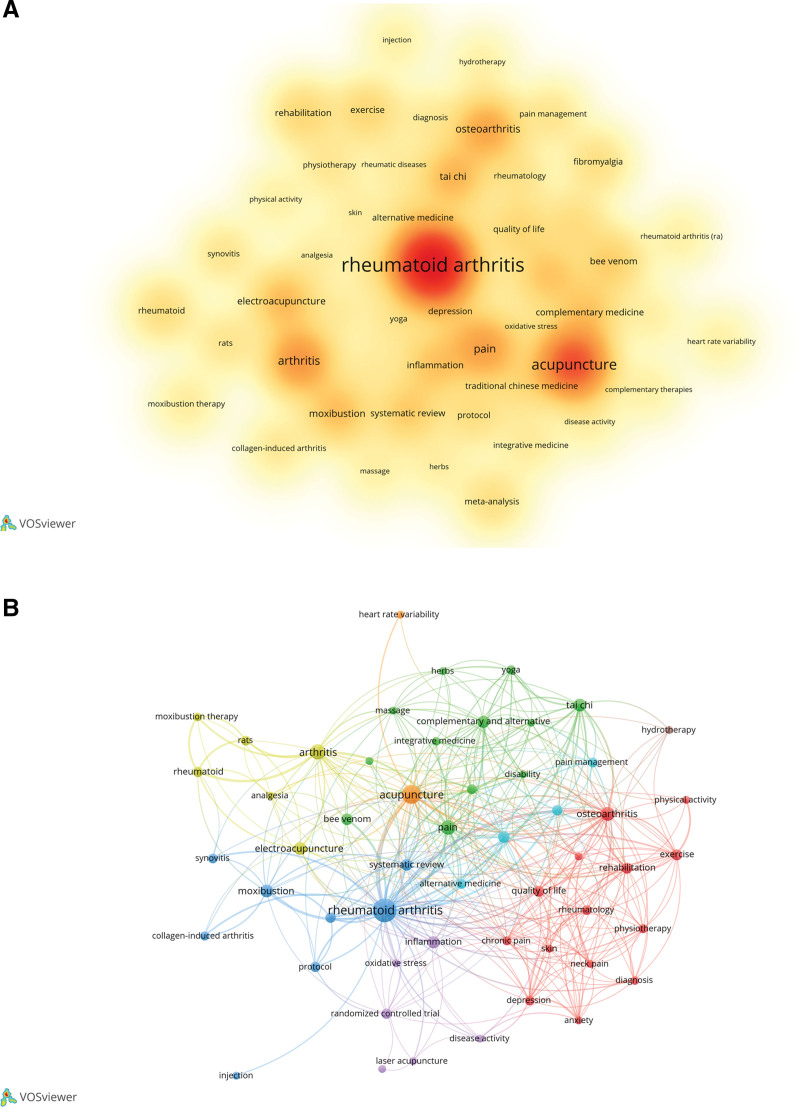
Analysis of keywords. (A) Visualization analysis of keyword density. (B) Cooccurrence analysis of keywords.

Keywords reflect the core and main contents of an article. Density reflects the frequency of keywords, whereas clustering effectively reflects research hotspots. In this study, 3675 keywords were obtained; with the top ten keywords being RA (149), acupuncture (72), arthritis (36), pain (30), osteoarthritis (28), Tai Chi (22), EA (21), moxibustion (20), complementary and alternative medicine (17), and inflammation (17) (Fig. 6A). The red cluster mainly included “osteoarthritis,” “rehabilitation,” “depression,” and “chronic pain,” revealing the role of nonpharmaceutical TCM therapies in improving the symptoms of RA. The green cluster was mainly composed of “complementary and alternative medicine,” “Tai Chi,” “massage,” and “yoga,” mainly showing the commonly used therapies. The blue cluster mainly included “rheumatoid arthritis,” “systemic review,” and “meta-analysis,” indicating the relevant research areas currently published on this topic (Fig. 6B).

### 3.8. Timeline viewer of keywords cluster

**Figure 7.**
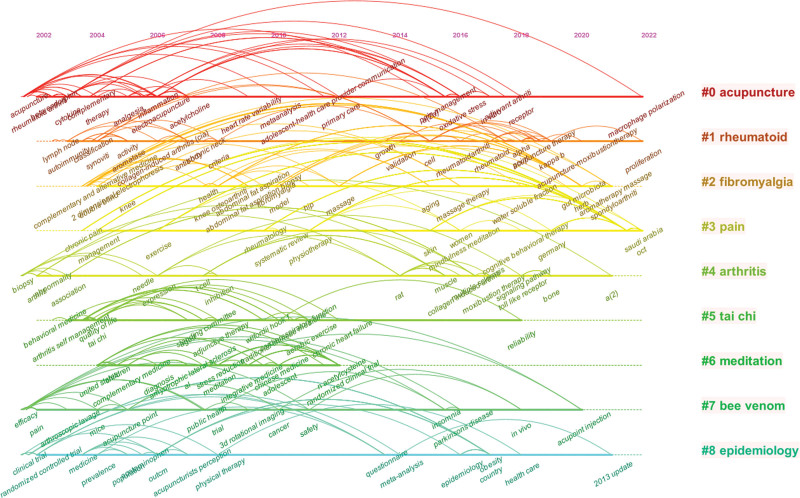
Visualization of timeline viewer of keyword clusters using CiteSpace.

In CiteSpace, a timeline is used to visually analyze the time span of each cluster and the relationships between different clusters, resulting in a keyword-clustering timeline (Fig. 7). Cluster numbers #0, #1, and #6 have a long time span, indicating that research on acupuncture and moxibustion treatment for RA has been ongoing for a long time and remains a hotspot in recent years. The timeline spanned from approximately 2018 to 2020, with numbers #4, #5, #6, and #7 indicating a gradual decline in research on the treatment of RA with Tai Chi and meditation around 2018.

### 3.9. Keyword clusters and citation bursts analysis

**Figure 8.**
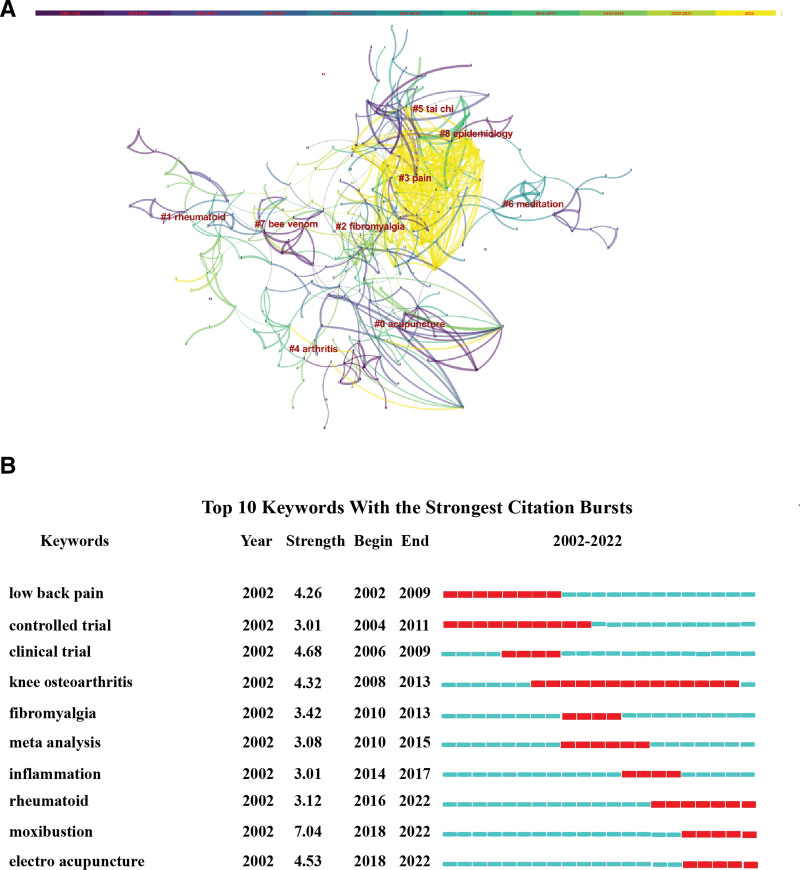
Visualization of keyword clusters and citation bursts analysis using CiteSpace. (A) Keyword clusters. (B) Citation bursts analysis.

The log-likelihood-ratio algorithm in CiteSpace was used to cluster keywords from the literature, and 9 main clusters were obtained (Fig. 8A). Cluster #0, acupuncture; #5, Tai Chi; #6, meditation; and #7, bee venom were nonpharmaceutical TCM therapies. Clusters #1, rheumatoid; #2, fibromyalgia; #3, pain; and #4, arthritis referred to the improvement of RA symptoms through nonpharmaceutical TCM therapies. Cluster 8, epidemiology focused on the epidemiological characteristics of RA.

Figure 8B presents the top ten keywords with the strongest citation bursts. Studies on RA over the last 20 years are roughly divided into 3 categories: (1) keywords such as low back pain, knee osteoarthritis, and fibromyalgia indicated that studies focused on RA symptom improvement by nonpharmaceutical TCM therapies from 2002 to 2013; (2) keywords such as meta-analysis, controlled trial, and clinical trial indicated that these studies emphasized on evaluating the efficacy of nonpharmaceutical TCM therapies in RA treatment from 2004 to 2015; and (3) keywords such as moxibustion and EA indicated that recent hot research directions have focused on the selection of nonpharmaceutical therapies for RA.

## 4. Discussions

With the development of big data, it is important for researchers and clinicians to understand the research progress in nonpharmaceutical TCM therapies for RA. Bibliometric analysis uses visualization software, such as VOSviewer, to comprehensively analyze the extant literature, understand research trends, and predict future hotspots.^[23]^ This study was the first bibliometric analysis of nonpharmaceutical TCM therapies for RA over the past 20 years.

### 4.1. Basic information

In this study, we conducted a bibliometric analysis of 567 publications on nonpharmaceutical TCM therapies for RA included in the WOS database between 2002 and 2022, and summarized the research topics and trends, hence presenting a global overview of this research area. From 2002 to 2022, the annual publication count on this topic was steadily increased, indicating rapid development and lasting research interest in this area. Notably, the People’s Republic of China and USA led the world in terms of publication count and total citations. In addition, 7 of the top ten academic institutions with the highest number of publications came from the People’s Republic of China.

In this study, we found that researcher Ernst E was the most productive author with the highest H-index, indicating his significant contribution to the field. After a thorough exploration of his articles, we found that he has performed many studies focusing on the objective evaluation of the clinical efficacy of nonpharmaceutical TCM therapies for RA, such as acupuncture, moxibustion, and Tai Chi, through systematic evaluation.^[24–26]^ These studies have provided objective evidence-based evaluation methods that can be applied to nonpharmaceutical TCM therapies for RA.

The quartile (Q) of a journal category was obtained from journal citation reports for 2021. According to the statistics of the top ten most productive and cited journals, most were classified as Q3 or Q2. Although the number of papers published by the *Cochrane Database of System Reviews* was relatively small, the total number of citations was higher than that of most journals, indicating its importance in this field.

Keyword cooccurrence and clustering identified that the main research frontiers and hotspots in this field focused on specific methods and the efficacy of nonpharmaceutical TCM therapies for RA.

### 4.2. Hotspots and frontiers

#### 4.2.1. Selection of nonpharmaceutical TCM therapies.

Nonpharmaceutical TCM therapies mainly include traditional Chinese acupuncture, EA, moxibustion, cupping, tuina or massage, Guasha, embedding, qigong, Tai Chi, Wuqinxi, Baduanjin, and Yijinjing. Among these, acupuncture-related therapies are the most commonly used for the treatment of RA, which was consistent with the results of the citation burst analysis. Tam et al^[27]^ designed a randomized, prospective, double-blind, placebo-controlled trial to evaluate the effects of EA, traditional Chinese acupuncture, and sham acupuncture in 36 patients with RA. They showed that, compared with sham acupuncture, both traditional Chinese acupuncture and EA may serve as adjuncts by reducing the number of tender joints in patients with refractory RA. Another study evaluated the effect of EA as an adjuvant treatment to first-line medications on the levels of bone metabolism biomarkers and interleukin-17 in the peripheral blood of patients with RA.^[16]^ Compared with traditional acupuncture, EA effectively increased the serum levels of the bone metabolism markers carboxyterminal propeptide of type I procollagen, N-terminal-midfragment of osteocalcin, and bone-specific alkaline phosphatase and reduced the concentrations of the inflammatory marker β form of the C-terminal telopeptide of type I collagen, interleukin-17, C-reactive protein, and tartrate-resistant acid phosphatase-5b. Liao et al^[28]^ conducted a randomized controlled trial (RCT) using moxibustion for RA-related pain and found that it could effectively alleviate pain, improve clinical symptoms, and decrease the disease activity of RA. Likewise, Wang designed a pilot single-blind RCT to explore the effects of Tai Chi in adults with RA and found that Tai Chi improved functional class I and II RA.^[29]^ However, the findings of these clinical studies are preliminary and large-scale multicenter RCTs are warranted to confirm these conclusions.

#### 4.2.2. Measurement of clinical efficacy.

Characterized by refractory, repetitive, and disabling characteristics, RA results in the destruction of joints, tendons, muscles, connective and fibrous tissues, seriously influencing the quality of life of patients. After reviewing the literature, we determined that most nonpharmaceutical interventions improved certain symptoms or indices, but did not focus on the root cause. Casimiro et al^[30]^ performed a systematic review and evaluated the effects of traditional Chinese acupuncture or EA on objective and subjective measures of disease activity in patients with RA. They showed that EA may be beneficial in alleviating symptomatic knee pain in these patients 24 hours after treatment for up to 4 months, whereas no statistically significant difference was found with traditional Chinese acupuncture. Wan et al^[31]^ conducted a network meta-analysis of RCTs to compare the efficacy of acupuncture-related therapies in the treatment of RA. The network meta-analysis showed that in terms of improving the disease activity score-28, EA combined with DMARDs was the best combined therapy, whereas fire needle therapy or moxibustion combined with DMARDs was the best for alleviating pain and improving serological markers. Bernateck et al^[32]^ performed an RCT to compare the efficacy of auricular EA with that of autogenic training. They found that auricular EA significantly alleviated pain, reduced the erythrocyte sedimentation rate, and increased the serum concentration of tumor necrosis factor-alpha compared with autogenic training. Ahmed et al^[33]^ conducted a study to evaluate the efficacy of bloodletting cupping as complementary therapy for the management of RA. They observed that this therapy improved the clinical condition of patients with RA and exerted modulatory effects on innate (percentage of natural killer cells) and adaptive (soluble interleukin-2 receptor concentration) cellular immune responses when combined with conventional therapy. Several systematic reviews found that Tai Chi therapy was safe for patients with RA and could improve their quality of life but not their physical function or pain.^[10,34]^ Based on this literature review, we concluded that in clinically specific applications, appropriate treatment should be selected in accordance with the actual situation.

This study had some limitations. First, all data were obtained only from WOS, whereas other databases, such as PubMed, were not included. However, WOS is the most authoritative and comprehensive database to date and is commonly used for scientometric analysis.^[35]^ Second, a few related articles published in Chinese were excluded, which may have influenced our conclusions. Furthermore, owing to the rapid development of nonpharmaceutical TCM therapies for RA, related research is frequently updated. With an increasing amount of high-quality literature emerging, it is probable that hotspots and frontiers will change; therefore, timely updates are necessary.

## 5. Conclusions

Through a bibliometric analysis of nonpharmaceutical TCM therapies for the treatment of RA, this study evaluated literature-related information including different years, countries or regions, authors, institutions, and journals; analyzed the evolution of this topic over the past 20 years; and predicted possible future research. Our results showed that the selection of nonpharmaceutical TCM therapies and evaluation of their specific effects are frontier and hot topics of research. Exploring more effective nonpharmaceutical treatments and providing higher-quality evidence-based medical results are conducive to solving the problems encountered in the clinical treatment of RA. The findings of this study provide far-reaching guidance to researchers in this field and can help interested researchers find potential partners.

## Author contributions

**Conceptualization:** Kongfa Hu.

**Data curation:** Hongqin Yin, Yanhui Zhu, Ling Li.

**Formal analysis:** Xiaojun Sun, Ling Li.

**Funding acquisition:** Kongfa Hu.

**Investigation:** Jun Shen.

**Methodology:** Yanhui Zhu, Kongfa Hu.

**Visualization:** Xiaojun Sun, Hongqin Yin.

**Writing – original draft:** Xiaojun Sun.

**Writing – review & editing:** Kongfa Hu.

## Supplementary Material




