# Supplementary material for: Bibliometric and visualized analysis of nonpharmaceutical TCM therapies for rheumatoid arthritis over the last 20 years using VOSviewer and CiteSpace software
Source: Medicine (Baltimore). 2023 Sep 29;102(39):e35305. doi: 10.1097/MD.0000000000035305 (PMC10545214; doi:10.1097/MD.0000000000035305)
Supplement: Supplementary file 1 [file medi-102-e35305-s001.docx]

**Supplemental Table 1**

Search strategy for Web of Science Core Collection database.

| **Set** | **Search query** |
| --- | --- |
| *#1* | TS= (“moxibustion” OR “acupuncture” OR “electroacupuncture” OR “auricular points” OR “needle” OR “needling” OR “embedding” OR “acupoint” OR “cupping” OR “guasha” OR “massage” OR “Qigong” OR “Tuina” OR “Tai Chi” OR “Wuqinxi” OR “Baduanjin” OR “Yijinjing”) |
| *#2* | TS= (“Rheumatoid arthritis” OR “RA”) |
| *#3* | (#1) AND (#2) |
