# Supplementary material for: Bibliometric and visualized analysis of nonpharmaceutical TCM therapies for rheumatoid arthritis over the last 20 years using VOSviewer and CiteSpace software
Source: Medicine (Baltimore). 2023 Sep 29;102(39):e35305. doi: 10.1097/MD.0000000000035305 (PMC10545214; doi:10.1097/MD.0000000000035305)
Supplement: Supplementary file 2 [file medi-102-e35305-s002.docx]

***Citation*** ***analysis of countries and institutions***

Citation represents the number of times an article has been cited by other articles and can be used as an indicator to reflect the influence and importance of the literature. In the distribution of countries with cited literature, USA, England, South Korea, and China were in top 4, with publications cited more than 1000 times. This indicated that research on nonpharmaceutical TCM therapies for rheumatoid arthritis conducted by researchers in these countries is more likely to be paid great attention from researchers in other countries (Supplemental Figure 1A). Supplemental Figure 1B showed the top 40 countries with the most citations and their collaborative networks. Among them, articles published from Kyung Hee Univ, Univ Maryland, and Harvard Univ had the most citations, which were all more than 500 times. This indicated that publications from these countries have important reference in the study of nonpharmaceutical TCM therapies for rheumatoid arthritis.

*
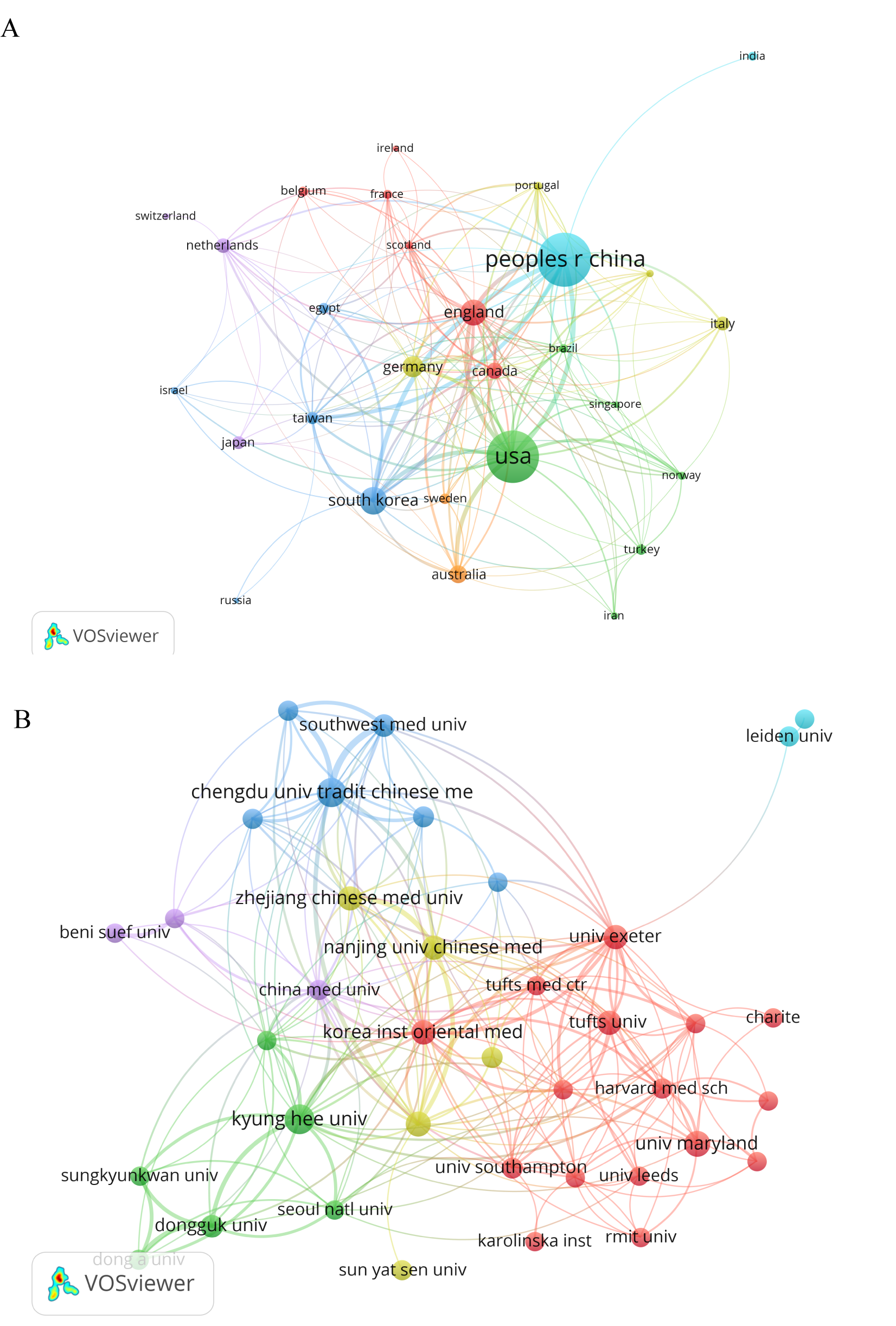
*

**Supplemental Figure 1.** Visualization of citation analysis of countries and institutions using VOSviewer. (A) Citation analysis of countries. (B) Citation analysis of institutions.
